# Porous Silicon Photonic Crystals Coated with Ag Nanoparticles as Efficient Substrates for Detecting Trace Explosives Using SERS

**DOI:** 10.3390/nano8110872

**Published:** 2018-10-23

**Authors:** Furu Zhong, Zhaofeng Wu, Jixi Guo, Dianzeng Jia

**Affiliations:** Key Laboratory of Energy Materials Chemistry, Ministry of Education, Key Laboratory of Advanced Functional Materials, Autonomous Region, Institute of Applied Chemistry, Xinjiang University, Urumqi 830046, Xinjiang, China; zhfuru@shzu.edu.cn (F.Z.); wzf911@mail.ustc.edu.cn (Z.W.); jxguo1012@163.com (J.G.)

**Keywords:** porous silicon photonic crystals, Ag nanoparticles, optical properties, SERS.

## Abstract

Picric acid (PA) is an organic substance widely used in industry and military, which poses a great threat to the environment and security due to its unstable, toxic, and explosive properties. Trace detection of PA is also a challenging task because of its highly acidic and anionic character. In this work, silver nanoparticles (AgNPs)-decorated porous silicon photonic crystals (PS PCs) were controllably prepared as surface-enhanced Raman scattering (SERS) substrates using the immersion plating solution. PA and Rhodamine 6G dye (R6G) were used as the analyte to explore the detection performance. As compared with single layer porous silicon, the enhancement factor of PS PCs substrates is increased to 3.58 times at the concentration of 10^−6^ mol/L (R6G). This additional enhancement was greatly beneficial to the trace-amount-detection of target molecules. Under the optimized assay condition, the platform shows a distinguished sensitivity with the limit of detection of PA as low as 10^−8^ mol/L, the linear range from 10^−4^ to 10^−7^ mol/L, and a decent reproducibility with a relative standard deviation (RSD) of ca. 8%. These results show that the AgNPs-modified PS PCs substrates could also find further applications in biomedical and environmental sensing.

## 1. Introduction

In recent years, ultra-sensitive detection of trace amounts of explosive chemicals has attracted widespread research attention due to the importance of these chemicals in many practical applications, including terrorist attacks, homeland security, and environmental protection [1,2,3,4,5]. Most explosives have low room-temperature vapor pressure; for example, the saturated vapor pressure of 2,4,6-trinitrotoluene (TNT), 2,4-dinitrotoluene (DNT), and picric acid (PA) are 9, 411, and 0.97 ppb, respectively [6]. Therefore, it is difficult to detect explosives quickly and accurately using gas sensors. Ion mobility spectrometry technology has distinct advantages in detecting volatile compounds and is often used to detect trace amounts of some explosives quickly and accurately [7]. Nevertheless, the poor selectivity and poor quantitative analysis hinder further applications of this technology. There has been a consequent demand for a simple, selective, and sensitive quantitative analysis method for the detection of trace explosives [8]. Surface-enhanced Raman spectroscopy (SERS), as a powerful chemical and biological analytical technique, has received much attention from researchers owing to its high surface sensitivity and molecular specificity [9,10]. Some researchers have used this technology to detect trace explosives [11,12,13,14].

Over the last few decades, owing to its unique optical and physical characteristics, porous Si has been widely used as sensors, such as biological sensors [15], electrochemical sensors [16], and optical sensors [17,18]. Among these, SERS-based sensors have received much attention from researchers [18,19,20]. The Raman enhancement effect in these sensors, which depends largely on the type and morphology of SERS substrate, can be attributed to electromagnetic (EM) and chemical mechanisms (CM) [21]. At some sites on a molecule, which can be referred to as “hotspots”, the local electric field becomes very strong; this leads to strong Raman signals from the molecules at these spots. Both the morphology and the substrate materials significantly influence the SERS effect. Noble metals, such as Au and Ag, even Au and Ag alloys, are the most widely used materials in this regard. On the other hand, many researchers have improved upon and optimized the substrate structure to obtain more and stronger electromagnetic hotspots by using sharp edges or tips and porous nanoparticles of these noble metals [3,22]. For example, porous Si coated with Au/Ag nanoparticles has been reported to obtain excellent SERS performance [1,19]. However, most researchers focus on the main SERS effect of single-layer porous Si structures; on the other hand, sensors based on the SERS effect of porous Si photonic crystals are still rarely reported. Particularly, to the best of our knowledge, the application of these sensors in explosives detection has not yet been reported. The photonic crystal structure can enhance the SERS effect of the analyte [23,24]. Unfortunately, most SERS-based sensors are not compatible with existing integrated circuit technology; the preparation process is also more complex. This hinders further application of SERS-based sensors. Porous Si is a sponge-like silicon material with very large specific surface areas, which is often prepared by anodic electrochemical etching. In recent years, porous Si has been widely used in many applications because of its excellent photoelectric properties and chemical properties, such as biological sensors [17,25], solar cell [26], biomedical devices [27], and catalysis [28]. The porosity of the material is controlled by varying the anodization current density, and the pore length is controlled by varying the time of etching for a certain etchant at constant temperature [29]. The pore diameter and surface morphology can also be tuned by varying the etching parameters and the Si wafer type. Thus, the preparation of porous silicon by anodic electrochemical etching is simple, and the repeatability of the process is very good [30]. Thus, this method is very suitable for the preparation of porous Si photonic crystals (PS PCs).

**Scheme 1:**
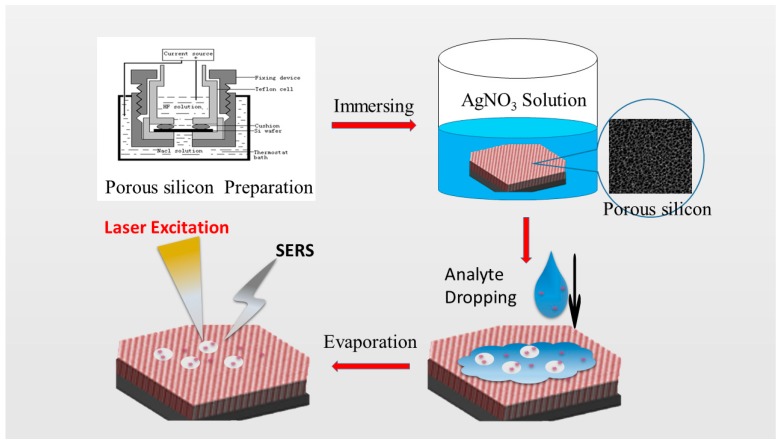
Schematic fabrication of silver nanoparticles incorporated porous silicon photonic crystals (PS PCs) for surface-enhanced Raman scattering (SERS).

In this study, the porous Si photonic crystals modified by Ag nanoparticles (AgNPs) as a SERS substrate were designed for qualitative identification and quantitative determination of trace amounts of R6G and PA explosives by the SERS method (Scheme 1). Porous Si photonic crystals were obtained by an anodic electrochemical etching method. AgNPs were prepared on PS PCs by a simple, controllable immersion plating technique. Compared with single-layer porous Si, the enhancement factor of the PS PC substrate is increased 3.58 times at the R6G concentration of 10^−6^ M. This enhancement was greatly beneficial for the trace-amount-detection of target molecules. Under optimal conditions, the observed detection limit of the explosive PA was estimated to be as low as 10^−8^ mol/L. Moreover, a satisfying linear relationship (*R*^2^ = 0.9667) in the concentration range between 1.0 × 10 ^−7^ and 1.0 × 10^−4^ mol/L was obtained.

## 2. Materials and Methods

### 2.1. Materials and Reagents

AgNO_3_ (99.8%), hydrofluoric acid, PA, and alcohol were purchased from Sinopharm Chemical Reagent Co., Ltd. (Shanghai, China) Rhodamine 6G was purchased from Shanghai Aladdin Bio-Chem Technology Co., Ltd. (Shanghai, China) Monocrystalline Si was purchased from the Tianjin Institute of Semiconductor Technology (Tianjin, China). All the chemicals were of analytical grade and were used without further purification. Wahaha pure water (deionized water) (TDS ≈ 0) was used for all the preparations.

### 2.2. Preparation of Porous Silicon Photonic Crystals

The highly-doped crystalline silicon (c-Si) p-type wafers with a resistivity of 0.02 to 0.06 Ω·cm were first cleaned ultrasonically several times in ternate baths of alcohol and acetone, before being dried in a nitrogen environment. Porous Si was prepared using an anodic electrochemical etching method, as reported in our previous papers [31]. In brief, the process was conducted in a homemade double cell etch tank, as illustrated in Scheme 1. The electrolyte was a mixture of aqueous HF (48% by weight) and alcohol (99.7% by volume) in a concentration ratio of 1:1. Current densities of 230 mA/cm^2^ for 1 s and 130 mA/cm^2^ for 1.5 s were applied alternately for the fabrication of multilayer porous silicon. The total etching time was 12 cycles. After etching, the samples were repeatedly washed with pure alcohol and distilled water immediately. Then, the porous Si samples were placed in an alcohol solution for subsequent use.

### 2.3. In Situ Synthesis of Silver Nanoparticles on Porous Silicon Photonic Crystals

Ag deposition on the PS PC samples was performed by using an immersion plating solution. Due to the abundant Si–H bonds on the porous silicon surface, Ag^+^ ions can be easily reduced to AgNPs. Thus, the AgNPs obtained without any chemical contamination present a great advantage due to the simplicity of operation and the fact that no toxic chemical reagents are introduced. Therefore, the immersion plating method is a simple, popular way to produce AgNPs on a substrate. Since the thickness of AgNPs can be adjusted by setting the immersion plating time, the longer the immersing time, the greater the yield of AgNPs is. AgNP arrays were prepared by using immersion plating solutions, according to the procedure reported by Zeiri et al. [32]. In brief, the 1 mM AgNO_3_ precursor solution was prepared in an alcohol solvent. The PS PC samples were completely immersed in the AgNO_3_ solution and then dried in air. In this study, five types of samples were obtained at five different immersion plating times: 30, 45, 60, 75, and 90 s. The AgNP-decorated PS PCs were washed with Wahaha pure water before SERS applications.

### 2.4. Characterization

Morphological and structural investigations of PS PCs were respectively performed using a field-emission scanning electron microscope (FESEM, Hitachi S-4800, Hitachi, Tokyo, Japan) and a Bruker D8 Advance Diffraction diffractometer (Bruker AXS, Karlsruhe, Germany) in the 2θ range from 10° to 80°, with Cu Kα radiation (λ = 0.15405 nm) at 40 kV and 40 mA. The reflectance spectra of the PS PC samples were obtained at room temperature by using a Hitachi U-4100 spectrometer (Hitachi, Tokyo, Japan).

### 2.5. Raman Measurement

SERS spectra were obtained using a HORIBA micro-Raman spectrometer (HORIBA Jobin Yvon, Kyoto, Japan) equipped with 50-mW Nd:YAG laser of 532-nm light and He–Ne laser of 633-nm light as excitation radiation. Low laser power of 1 mW was used to avoid sample heating effects, and the laser spot focused on the sample surface was about 2 μm in diameter. The SERS spectra of R6G were accomplished with an excitation wavelength of 633 nm, while the SERS spectra of PA were accomplished with an excitation wavelength of 532 nm. The data acquisition time was 3 s for each accumulation. A drop of (10 µL) of the R6G or PA alcoholic solution (analyte) was pipetted out onto the PS PC substrate and was dispersed to a circular shape area ~0.5 cm^2^. The same sample preparation method was used for all concentrations of R6G and PA. The reproducibility was evaluated at 5 randomly chosen spots on the PS PC substrate. The Si–Si Raman peak of 521 cm^−1^ was used to calibrate the spectrometer.

## 3. Results and Discussion

### 3.1. Structure and Morphological Characteristics

**Figure 1:**
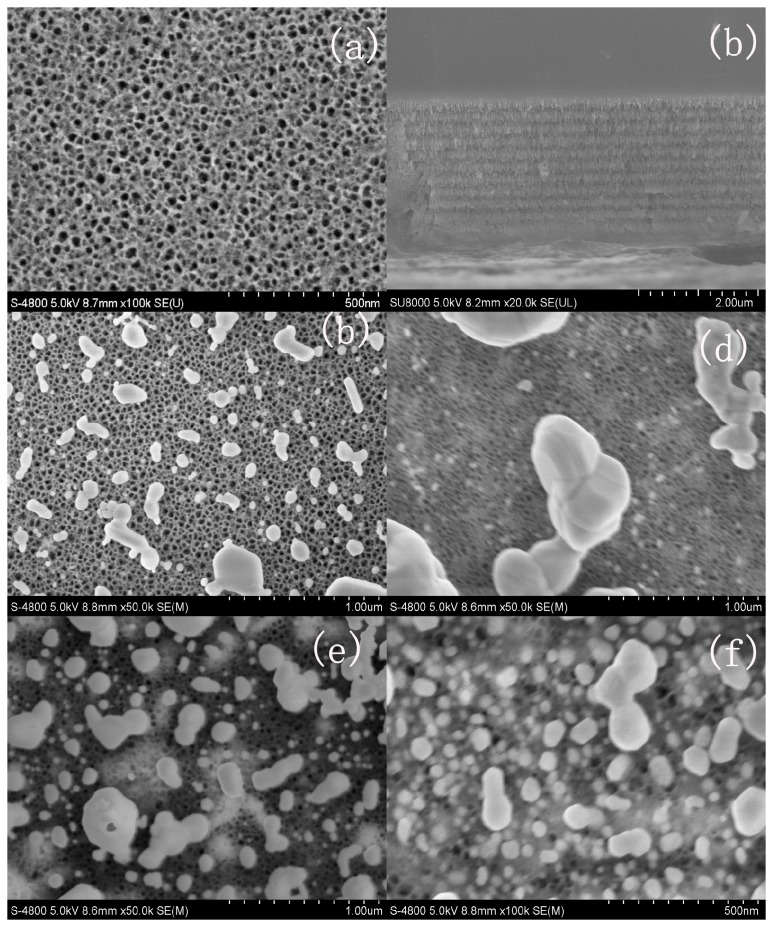
Scanning electron microscope (SEM) images of PS PC substrates (**a**) top view of porous silicon; (**b**) cross-section view of porous silicon; (**c**–**f**) surface topographies of silver nanoparticles (AgNP)-decorated porous Si with immersing times of 30, 45, 60, and 75 s, respectively.

Figure 1 shows the SEM images of the surface and cross-section of the PS PCs. The surface structures of the freshly prepared PS PCs are shown in Figure 1a. Top-view observation revealed that the PS PCs have a uniform distribution of pores with a diameter of 20 to 50 nm, estimated from the SEM images. The cross-section SEM images, as shown in Figure 1b, showed the hierarchy of 12 cycles and that the thickness of porous Si was ~2 µm. The layers with high and low porosities were formed by anodic electrochemical etching using large and small current densities, respectively. Figure 1c–f shows the AgNP-decorated porous Si obtained with different soaking times (30–75 s). These images revealed that with increasing soaking time, the number of AgNPs increased and the gap between the nanoparticles reduced. After soaking for more than 75 s, many overlapping AgNPs were observed, as shown in Figure 1f.

### 3.2. Reflectance and Raman Spectrum

**Figure 2:**
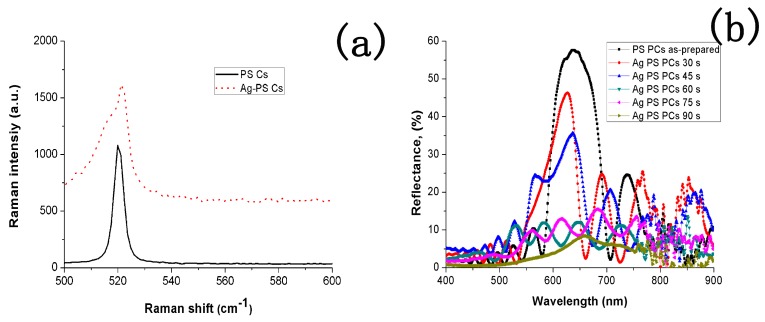
(**a**) Raman pattern and (**b**) reflectance spectra of AgNP-decorated PS PC samples with different Ag deposition times. The image shows the behavior of the reflectance spectrum of the PS PC samples with and without Ag nanoparticles.

Samples previously analyzed by FESEM were subjected to specular spectral reflectance measurements to check the optical response of the Ag-PS nanostructures. We have presented in Figure 2b the frequency evolution corresponding to Ag deposition time ranging from 30 to 90 s. With increasing Ag deposition time, the photonic bandgap gradually disappeared. This may be attributed to the increase in AgNP concentration and the surface becoming rougher. This observation also reveals the presence of penetration of the porous silicon. However, the existence of interference fringes was observed up to an Ag deposition time of 75 s. When the deposition time was 90 s, almost no interference effect was observed. These results are in good agreement with the SEM results. Figure 2a shows the Raman spectra of the PS PC samples with and without silver nanoparticles coating. It can be seen that both types of samples showed a Raman peak at 520 cm^−1^. The symmetrical Raman peak indicated almost no stress change in the as-prepared PS PCs. However, the AgNP-decorated samples show an asymmetry in the low-frequency side, compared to crystalline Si; this downshift of the phonon frequency was related to the width of the pores [20]. Redshift and broadening of absorption peaks were also observed. The shifts in absorption band and asymmetry can be attributed to a quantity-size effect and the surface effect [33], while the peak broadening was attributed to an increase in local temperature [34]. These results indicate that the local temperature is more likely to increase on AgNP-decorated porous Si.

### 3.3. Raman Scattering

As is well known, SERS is a powerful fingerprint analytical technique to provide real-time onsite detection for biological and chemical sensing owing to its advantageous non-destructive and non-invasive nature [7]. Here, we use R6G and PA as Raman probes to investigate the SERS-active substrate, whose Raman-active modes are summarized in Table 1.

**Figure 3:**
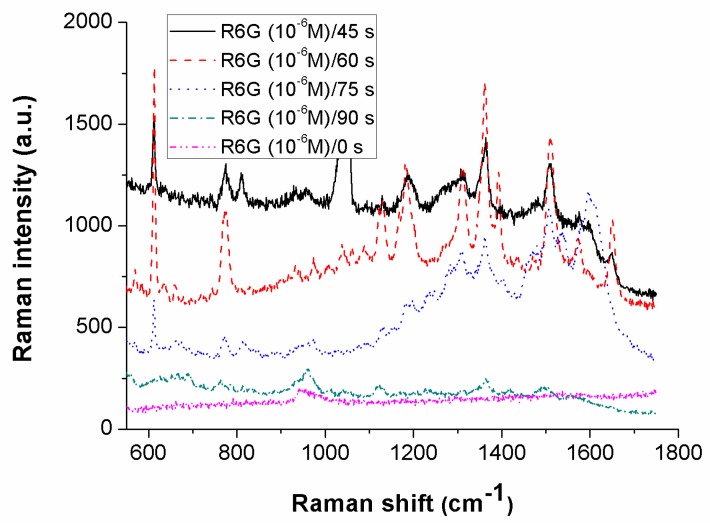
SERS spectra of R6G molecules (10^−5^ mol/L) on AgNP-decorated PS with different immersing times.

It is known that AgNP size depends on the deposition time. Thus, the optimal immersing time can be obtained experimentally. Figure 3 illustrates the comparative SERS intensities of R6G molecules (10^−5^ mol/L) obtained on PS PC samples. The typical bands of R6G were observed at 612, 772, 1087, 1127, 1189, 1312, 1363, 1509, and 1651 cm^−1^ in the first four cases, which were well indexed to the C–C in-plane bending in xanthene rings, C–H out-of-plane bending, C–H in-plane flexural stretching vibration, C–H out-of-plane vibration in xanthene rings, hybrid-mode associated with the NHC_2_H_5_ group and xanthene rings, and C–C ring stretching to xanthene rings, respectively [19,37]. When the deposition time/immersing time exceeded 90 s, it was difficult to observe the Raman peaks of R6G because the layer of AgNPs was too thick, which hindered the electromagnetic interaction between Si and AgNPs [38]. The SEM image, shown in Figure 1f, confirms this result. The results show that the sample prepared with an immersing time of 60 s has the largest Raman enhancement effect.

**Figure 4:**
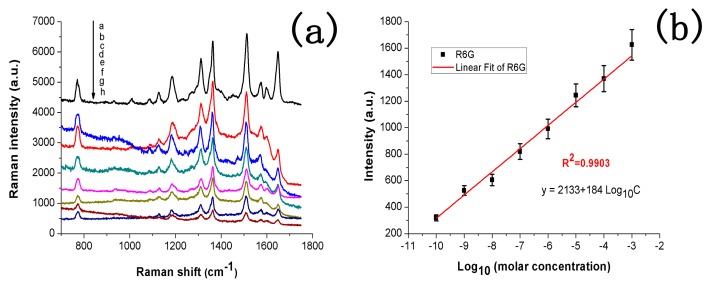
(**a**) SERS signals of R6G, at different concentrations ranging from 10^−3^ to 10^−10^ mol/L, absorbed on AgNP-decorated PS PC samples. (**b**) Corresponding linear fitting of Raman intensity at 1512 cm^−^^1^ with logarithmic R6G concentration. Error bars present the standard deviation obtained from three independent measurements. (λ_excitation_ 633 nm, acquisition time 3 s, laser power 1 mW).

Figure 4 shows the SERS signals of R6G (concentration 10^−3^–10^−10^ mol/L) adsorbed on the AgNP-decorated PS PC samples, upon excitation at 633 nm. Although the Raman intensity largely decreased upon reducing the R6G solution concentration, the main characteristics of R6G molecules can be identified even when the solution concentration was as low as 10^−10^ mol/L. A 10-μL reference sample with 10^−5^ mol/L R6G deposited on a glass slide was measured. The constructive Raman band at 1512 cm^−1^ was selected to estimate the SERS enhancement factor (EF) based on the following Equation 1 [39]:

*EF* = (*I*_SERS_/*N*_NR_)/(*I*_NR_/*N*_SERS_） (1)

where *I*_SERS_ is the SERS intensity of R6G on PS PCs and *I*_NR_ is the normal Raman intensity of R6G at the same band. *N*_NR_ and *N*_SERS_ represent the corresponding numbers of molecules in the focused incident beam on the substrate, which can be estimated using a reported method [40]. In our experiments, the focus area of the laser beam is ~4 μm^2^. A 10-μL drop of R6G (10^−5^ mol/L) contains 6 × 10^6^ molecules. Thereby, the EF was calculated to be 4.3 × 10^6^. The EF for AgNPs decorated on monolayer porous silicon (PS) is 1.2 × 10^6^. SERS results demonstrated that nanoparticles with a molecular-dimension gap between the particles led to significant enhancement in Raman scattering of the linked molecules and nanoparticles. A linear dependence was observed between the logarithmic concentrations of R6G and the intensities of the fingerprint peaks (1512 cm^−1^), see Figure 4b, as per the following equation:

*y* =2133 +184lg*C* (2)

where *y* is the peak intensity of the SERS spectra of R6G and *C* is the R6G concentration. Therefore, it can be concluded that AgNP-decorated PS PCs can be applied as sensitive SERS substrates for the quantitative detection of trace R6G. It can also be seen that the results show excellent linearity (*R*^2^ = 0.9903) over a wide concentration range.

**Figure 5:**
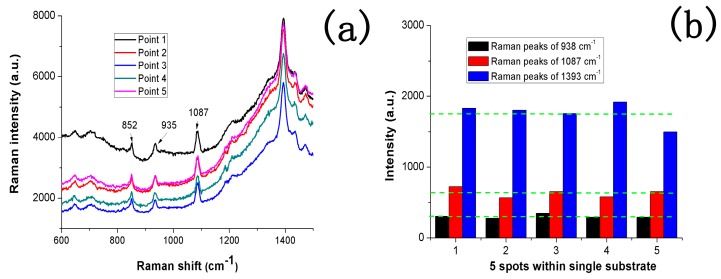
(**a**) SERS spectra of PA (10^−5^ mol/L) adsorbed on AgNP-decorated PS PC substrate, obtained from five random spots; (**b**) The transverse dash line shows the average SERS intensity of the five random spots within single substrate.

The AgNP-decorated PS PC nanostructures were used as SERS substrates to explore their application in quantitative analysis of trace PA. As is well known, PA is a common explosive that is widely used in the manufacture of acid dyes, photographic drugs, explosives, and pesticides, etc. Thus, PA seriously pollutes the environment through water and soil [41]. Here, PA was used as the target analyte to effectively verify the performance of the PS PC substrate. A 10-μL solution of a particular concentration was drop-casted on the PS PC substrate. With regard to the significant issue of reproducibility, we first observed the uniformity of the substrate. Figure 5 shows that the SERS signals were all of comparable intensity, indicating that the PS PC substrates exhibited uniform SERS enhancements over their entire surface. For the intensities of the characteristic peaks at 938, 1087, and 1393 cm^−1^ of PA in these five spectra, which were obtained at randomly selected spots under identical experimental conditions, the relative standard deviations (RSD) were 8.16, 8.09, and 8.05%, respectively. This is probably due to the uniform coating of AgNPs on PS PC nanopore structures.

**Figure 6:**
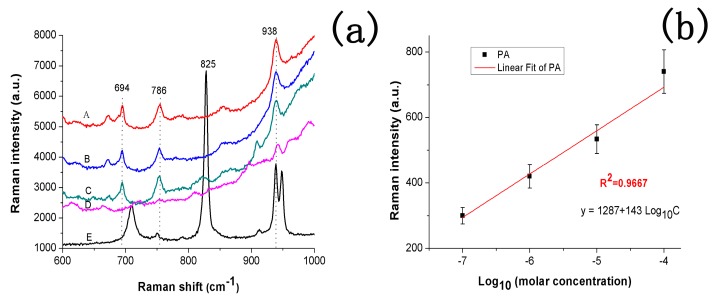
(**a**) SERS signals of PA adsorbed at different concentrations on AgNP-decorated PS PC substrates: (A) 10^−4^ mol/L, (B) 10^−5^ mol/L, (C) 10^−6^ mol/L, (D) 10^−7^ mol/L, (E) solid PA and (**b**) the relationship between the Raman intensity (938 cm^−1^) and PA concentrations.

Figure 6a shows the SERS spectra for PA detection. The Raman intensity decreased upon reducing the PA concentration from 10^−4^ to 10^−7^ mol/L. The characteristic peaks, see Table 1, of PA at 786 cm^−1^, 938 cm^−1^ (ring breathing), and 1085 cm^−1^ (phenolic C–O stretching) were observed at all the concentrations tested. Compared with the Raman characteristic peaks of PA itself, the Raman peaks of the PS PC substrates showed some redshift, attributed to the stress on the contact surface between silicon and silver [42].

Figure 6b shows the plot of SERS intensity as a function of PA concentration (at 938 cm^−1^) against explosive concentration on a logarithm scale. A relatively good linear response was obtained at PA concentrations ranging from 10^−4^ to 10^−7^ mol/L. The linear regression equation was *y* = 1287 + 143lg*C*, with a correlation coefficient (*R*^2^) of 0.9667, where *y* is the intensity of the Raman peak at 938 cm^−1^ and *C* is the PA concentration. The standard deviation (*SD*) of the intercept is 85.66, and the *SD* of the slope is 15.26. The limit of detection of PA (*S*/*N* = 3) was 10^−8^ mol/L. Compared with the reported data summarized in Table 2, the results obtained herein indicated that PS PCs could be used as a potential substrate for SERS-quantitative detection.

This SERS platform showed high sensitivity, low detection limit, and wide linear range, which can be ascribed to the PS PC substrate. In general, SERS enhancement in the Raman signal from AgNP-decorated PS PCs is mainly correlated to the electromagnetic enhancement mechanism [43]. However, in this case, chemical enhancement may have played an important role in SERS enhancement. Porous Si is a new functional material with a sponge-like structure, based on nanocrystalline Si particles. Thus, this nano-dimensional semiconductor structure is very conducive to charge transfer between semiconductor nanoparticles and the target molecules [44]. The Si nanoparticles in the present AgNP-decorated PS PCs nanostructure may lead to enhanced electron charge transfer from the AgNPs to the target molecules, which in turn affect the SERS enhancement. For the PS PC nanostructure, the internal reflections in multilayers can enhance the Raman signals [45]. Compared to monolayer porous Si or glass substrate, PS PC substrates have an extraordinary scattering effect in response to the exciting light, and thus, it has a stronger photoelectric coupling effect. As shown in Figure 6, the AgNP-decorated PS PC substrates have a wider linear dynamic range (LDR) owing to them having a higher surface-to-volume ratio than porous silicon [46]. As is known, SERS intensity depends on hotspots. The high surface-to-volume ratio implied that the substrate has a larger surface area, which greatly increased the hotspot area and led to wide LDR.

## 4. Conclusions

In summary, we have presented new PS PC SERS substrates with a molecule concentrating effect by integrating AgNPs into the micropores of PS PCs. The AgNP-decorated PS PCs were prepared by an in-situ method by immersion in an AgNO_3_ solution. The experimental results showed that this substrate has a sufficiently high detection sensitivity for the easy detection of R6G at concentrations as low as 10^−10^ mol/L by a simple dipping method, and its enhancement factor reaches 1.2 × 10^6^ at the concentration of 10^−6^ mol/L. The detection of a biomolecule, e.g., PA, was also explored. A good linear response was obtained for PA concentrations ranging from 10^−4^ to 10^−7^ mol/L and the detection limit was as low as 10^−8^ mol/L. More importantly, the RSD values of the Raman signals were ~8%. Thus, the as-prepared nanostructures can be used to detect trace compounds with minimal analyte consumption using PS PC substrates.

## Figures and Tables

**Table 1:** Assignment of vibrational modes in SERS Spectra of Rhodamine 6G dye (R6G) and picric acid (PA).

PA Raman Shift (cm^−1^)	Assignment [3]	R6G Raman Shift (cm^−1^)	Assignment [35,36]
825	C–H bending	612	C−C ring in-plane bending in xanthene/phenyl rings
938	ring breathing	772	C−H out-of-plane bending
1088	phenolic C–O stretching	797	hybrid mode (xanthene/phenyl rings and NHC_2_H_5_ group)
1330	C–C stretching	1127	C−H in-plane bending in xanthene/phenyl rings
1335	NO_2_ symmetric stretching	1187	C−H in-plane bending in xanthene ring
1564	C–NO_2_ asymmetric stretching	1204	hybrid mode (xanthene/phenyl rings)
		1275	C−O−C stretching in COOC_2_H_5_ group on phenyl ring
		1312	hybrid mode (xanthene/phenyl rings and NHC_2_H_5_ group)
		1363	C−C stretching in xanthene ring
		1449	C−N stretching in NHC_2_H_5_
		1509	C−C stretching in xanthene ring
		1575	C−C stretching in phenyl ring
		1595	hybrid mode (phenyl ring with COOC_2_H_5_
		1651	C−C stretching in xanthene ring

**Table 2:** Summary of reported detection limits obtained using SERS technique for PA sensing.

Substrate	Detection Limit (mol/L)	Ref.
Positively charged silver nanoparticles	2.5 × 10^−5^	[11]
Versatile gold-based SERS substrates	10^−6^	[13]
Ag nanotriangles-loaded filter paper	10^−6^	[3]
Ag nanoparticles-loaded porous silicon	10^−8^	This work
